# A Complex Molecular Profile in Melanoma: Intratumoral Heterogeneity and Therapeutic Implications

**DOI:** 10.3390/ijms27219870

**Published:** 2026-11-04

**Authors:** Yinfei Tan, Giuliana G. Berardi, Tingwei Guo, Anjali Seth, Anthony J. Olszanski, Hong Wu, Y. Lynn Wang

**Affiliations:** 1 Department of Pathology, Fox Chase Cancer Center, Philadelphia, PA 19111, USA; 2 Department of Medical Oncology, Fox Chase Cancer Center, Philadelphia, PA 19111, USA; 3 Department of Pathology, Temple University, Philadelphia, PA 19140, USA; 4 Cancer Signaling and Microenvironment Program, Fox Chase Cancer Center, Philadelphia, PA 19111, USA

**Keywords:** melanoma, *BRAF*, *KIT*, *NF1*, *NRAS*

## Abstract

Complex molecular alterations in cancers influence tumor biology, therapeutic response, and resistance. We report a case of cutaneous melanoma demonstrating extensive intratumoral heterogeneity with important clinical implications. A 77-year-old man with stage IIA cutaneous melanoma developed an isolated nodal recurrence one year after resection. Comprehensive next-generation sequencing of the metastatic lesion revealed an uncommon genomic profile consisting of a class 3 *BRAF* Y472V mutation, two *NF1* loss-of-function mutations, and two concurrent *KIT* mutations: a dominant, potentially targetable K642E mutation and a low-frequency D816V mutation associated with resistance to tyrosine kinase inhibitors. The tumor exhibited a high tumor mutational burden and microsatellite stability. The patient received neoadjuvant nivolumab plus relatlimab followed by surgical resection, achieving complete pathologic response. Repeat sequencing of the resected specimen demonstrated complete molecular clearance of all previously detected variants. This case illustrates the value of comprehensive genomic profiling in revealing biologically complex melanoma with coexisting driver and resistance-associated alterations. The presence of a treatment-naïve *KIT* resistance mutation suggests preexisting subclonal heterogeneity that may influence future targeted therapy, while high tumor mutational burden and *NF1* loss may have contributed to the excellent response to immune checkpoint blockade.

## 1. Introduction

Melanomas predominantly arise from UV-damaged melanocytes in the basal layer of the epidermis. Constitutive activation of the receptor tyrosine kinase (RTK)-mitogen-activated protein kinase (MAPK) pathway underlies both melanoma initiation and progression. The key components of this pathway, including the upstream RTK (KIT), signal transducers (RAS and RAF) and negative regulator (NF1), are frequently mutated in melanoma, leading to sustained MEK-ERK activation, driving unregulated tumor cell survival and proliferation [1]. The Cancer Genome Atlas (TCGA) classified cutaneous melanoma into four genomic subtypes based on the dominant driver mutation: *BRAF*-mutant, *RAS*-mutant, *NF1*-mutant, and triple wild-type [2].

**Figure 1:**
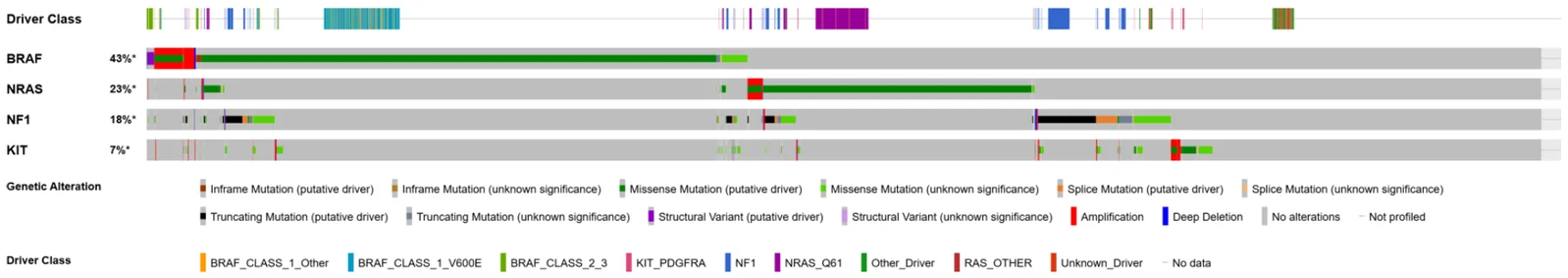
Relationship of commonly mutated genes in combined 28 studies of melanoma. Columns (vertical lines) show 4592 samples. Tracks display types of mutations in each gene indicated. A track of driver types is included at the top. Mutual exclusivity of strong gene mutations is apparent. Compiled and downloaded from cBioPortal for Cancer Genomics on 23 September 2026. * Not all samples are profiled according to cBioPortal.

*BRAF* mutations, which occur in approximately 40–50% of cutaneous melanomas, represent the most common oncogenic alterations in this disease (Figure 1). Based on in vitro biochemical differences, *BRAF* alterations have been classified into three classes (classes 1, 2, and 3), according to their kinase activity, dimerization status, and dependence on upstream RAS activity [2].

*BRAF* mutations at V600 including V600E/K/M are class 1 *BRAF* mutations. Substitution of the valine residue at position 600 destabilizes the inactive, autoinhibited conformation of the kinase domain, allowing class 1 mutants to signal with high kinase activity as active monomers [3]. These BRAF mutants mediate strong activation of downstream ERK independent of upstream RAS activation. For this class, *BRAF* V600E accounts for approximately 80–90% of cases in the setting of low chronic sun damage (CSD), whereas *BRAF* V600K, accounting for approximately 10–20% of class I mutants, is more frequently observed in melanomas with high CSD [2]. Tumors harboring Class 1 *BRAF* alterations are highly sensitive to combined BRAF and MEK inhibition [4]. A clinical trial showed a 67% overall response rate (ORR) to dabrafenib and trametinib combined therapy [5].

Class 2 *BRAF* variants are non-V600 activating mutations found in approximately 5–10% of *BRAF*-mutant melanomas. With elevated basal kinase activity, Class 2 mutants function as dimers and drive RAS-independent constitutive MAPK signaling. Therapeutically, standard monomer-selective BRAF inhibitors, such as vemurafenib, are largely ineffective against class 2 mutants [6].

Class 3 *BRAF* mutations also occur outside codon 600. Unlike class 1 and class 2 *BRAF* mutations, they represent a distinct, kinase-impaired or kinase-dead subset of BRAF mutants [7]. Class 3 mutant BRAF binds to and activates the catalytic function of wild-type CRAF, functioning as signaling “amplifiers” that require upstream RAS to activate the downstream MAPK pathway [8]. Clinically, melanomas harboring class 3 *BRAF* mutations are generally resistant to BRAF inhibitor monotherapy and exhibit limited responses to MEK inhibitor monotherapy [9,10]. However, these tumors may be sensitive to agents targeting upstream RAS activation or combined MAPK pathway blockade [7].

*NRAS* is the second most mutated gene seen in approximately 15–25% of cutaneous melanoma patients (Figure 1). RAS proteins are small intracellular GTPases that activate the MAPK pathway via downstream BRAF, as well as the phosphoinositide 3-kinase (PI3K)/AKT pathway [2]. Approximately 80–90% of mutations occur at codon 61 (Q61R/K/L), which abolishes GTP hydrolysis, rendering the protein constitutively active in a GTP-bound conformation, driving downstream signaling cascades [11]. Patients with *NRAS* mutations have shorter progression-free survival compared to their *BRAF* mutant counterparts, possibly due to dual activation through both MAPK and AKT signaling pathways. Strong activating *NRAS* and *BRAF* mutations are largely mutually exclusive (Figure 1) [12].

*NF1* is mutated in approximately 10–18% of melanomas, representing the third most frequently altered gene in melanoma (Figure 1). *NF1* encodes neurofibromin acting as a negative regulator of the MAPK pathway. It inactivates RAS by facilitating GTP hydrolysis to GDP [13]. *NF1*-mutant melanomas often exhibit biallelic loss. Loss-of-function mutations serve as key driver alterations, of which a substantial proportion are truncating mutations, such as nonsense variants. In the absence of functional NF1, RAS remains in its active, GTP-bound state for a prolonged period, leading to hyperactivation of the downstream RAS signaling pathway [12].

Therapeutically, patients with *NF1*-mutant melanoma do not demonstrate consistent response to MEK or PI3K inhibitors [14]. Interestingly, *NF1*-mutant melanomas are frequently associated with a high tumor mutational burden (TMB), which is generally predictive of an improved response to immunotherapy [2]. Furthermore, loss of *NF1* upregulates PD-L1 expression through a RAS-independent, NF-kappaB–dependent mechanism, thereby promoting immune evasion [15]. Consistent with both high TMB and PD-L1 upregulation, *NF1*-mutant melanomas demonstrate favorable clinical responses to anti-PD-1 immune checkpoint inhibitors [16]. Notably, *NF1* loss can co-occur with *BRAF* alterations, particularly non-V600 Class 2 and Class 3 variants (Figure 1) [17,18].

Finally, *KIT* mutations are the least common among the four genes, occurring in 7–15% of cases (Figure 1) [19]. KIT protein functions as an RTK. Since both class 1 BRAF and mutant KIT hyperactivate downstream signaling through the MAPK/ERK pathway, there is strict mutual exclusivity between *BRAF* V600 and *KIT* mutations (Figure 1) [20]. *KIT* mutations are predominantly localized to exons 11, 13, and 17. Mutations in exon 11, which encodes the juxtamembrane domain, account for ~70% of all *KIT* mutations in melanoma, with L576P being the most frequent hotspot mutation. Mutations in exon 13, Kinase Domain I, account for ~10–15%, with K642E as the most dominant variant. Mutations in other exons are less common, including extracellular domain alterations in Exon 9 and activation loop mutations in Exons 17 and 18 [21]. *KIT* alterations are typically enriched in “triple negative” melanomas for which KIT tyrosine kinase inhibitors (TKIs) have yet to be approved by the FDA [22]. Imatinib is the primary TKI evaluated in phase II clinical trials for *KIT*-mutant melanoma. It demonstrates an ORR of 20–30% with a modest median progression-free survival of ~3–4 months [23,24,25].

We report here a case of cutaneous melanoma harboring a rare genomic co-occurrence of a Class 3 *BRAF* mutation, two loss-of-function mutations of *NF1*, and dual *KIT* mutations which include a de novo TKI-resistant variant, highlighting the clinical significance of intratumoral heterogeneity.

## 2. Case Description

**Figure 2:**
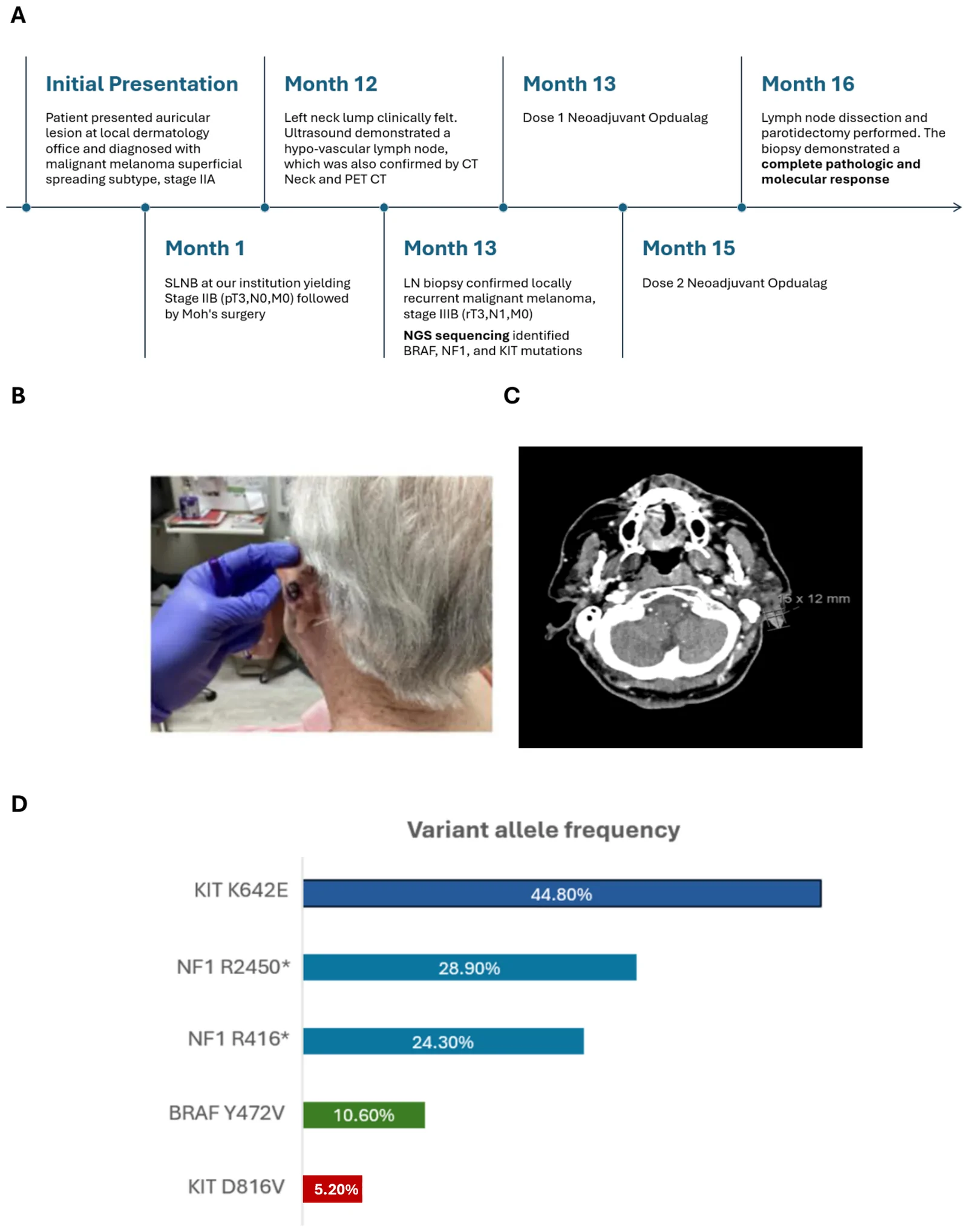
(**A**) Timeline of patient testing and treatment course; (**B**) left posterior auricular lesion. (**C**) Neck CT with contrast showing the left level 2A lymph node. (**D**) Variant allele frequencies of the driver mutations in the Month 13 lymph node biopsy (See Table 1 for the full list). * Indicates a nonsense mutation.

A 77-year-old man presented initially with a left posterior auricular lesion that was pruritic and non-healing (See disease course in Figure 2A). He had a history of basal cell carcinoma and squamous cell carcinoma. He reported significant sun exposure throughout his lifetime. He was a Vietnam War Veteran and worked for a gas company that required frequent outdoor site visits. He sought evaluation by his local dermatologist who noted the lesion to be black with irregular borders and clinically ulcerated in the center (Figure 2B). A shave biopsy was performed that demonstrated malignant melanoma, superficial spreading subtype, measuring 2.6 mm in depth with 6 dermal mitosis/mm^2^. Ulceration, vascular invasion, neurotropism, and satellitosis were absent, yielding clinical Stage IIA (T3a, N0, M0).

**Table 1:** Variants identified in patient’s lymph node biopsy.

Gene	Protein Change	cDNA Change	VAF (%)	Reference Transcript	Tier
ARID1B	p.Gly257fs	c.770delG	22.0	ENST00000346085	II
**BRAF**	**p.Tyr472Val**	**c.1414_1415delinsGT**	**10.6**	**ENST00000288602**	
CARD11	p.Arg187Gln	c.560G>A	31.7	ENST00000396946	
CBL	p.Ile361Ser	c.1082T>G	22.4	ENST00000264033	
CBLC	p.Trp221*	c.663G>A	37.4	ENST00000270279	II
CTCF	p.Pro159Leu	c.476C>T	30.4	ENST00000264010	
DNMT3A	p.Ser603Phe	c.1808C>T	29.2	ENST00000321117	
EPHA3	p.Glu615Lys	c.1843G>A	10.3	ENST00000336596	
ERBB2	p.Pro300Phe	c.898_899delinsTT	28.2	ENST00000269571	
ERBB2	p.Gly815Glu	c.2444G>A	26.2	ENST00000269571	
FAS	p.Ala301fs	c.900dupA	23.8	ENST00000355740	II
FGFR4	p.Ala394Val	c.1058-71C>T	25.2	ENST00000393637	
GATA3	p.Gly65Glu	c.194G>A	12.4	ENST00000379328	
GNAS	p.Asn77Ser	c.230A>G	27.4	ENST00000371100	
GNAS	p.Arg380Cys	c.1138C>T	27.8	ENST00000371085	
GRIN2A	p.Glu1301Lys	c.3901G>A	26.6	ENST00000396573	
HGF	p.Pro460Leu	c.1379C>T	35.9	ENST00000222390	
IKZF1	p.His480Tyr	c.1438C>T	10.0	ENST00000331340	
**KIT**	**p.Lys642Glu**	**c.1924A>G**	**44.8**	**ENST00000288135**	**I**
**KIT**	**p.Asp816Val**	**c.2447A>T**	**5.2**	**ENST00000288135**	**I**
KMT2C	p.Pro1739Ser	c.5215C>T	17.4	ENST00000262189	
LRP1B	p.Asp4377Asn	c.13129G>A	24.3	ENST00000389484	
LRP1B	p.Gly3654Glu	c.10961G>A	26.9	ENST00000389484	
LRP1B	p.Arg790Gln	c.2369G>A	19.1	ENST00000389484	
LRP1B	p.Glu630Lys	c.1888G>A	10.4	ENST00000389484	
LRP1B	p.Asp41Asn	c.121G>A	22.0	ENST00000389484	
MET	p.Thr582Ile	c.1745C>T	18.7	ENST00000318493	
**NF1**	p.Arg416*	**c.1246C>T**	**24.3**	**ENST00000358273**	**II**
NF1	p.His647Tyr	c.1939C>T	19.4	ENST00000358273	
**NF1**	p.Arg2450*	**c.7348C>T**	**28.9**	**ENST00000358273**	**II**
NF1	p.Thr2804Ile	c.8411_8412delinsTT	13.2	ENST00000358273	
PDGFRA	p.Glu386Lys	c.1156G>A	28.2	ENST00000257290	
PDGFRA	p.Leu809Ser	c.2426T>C	25.2	ENST00000257290	
PIK3R2	p.Pro721Leu	c.2162C>T	34.1	ENST00000222254	
POLE	p.Asn423fs	c.1269_1290delTCTCAA GGCGGCCGCCAAGGCC	27.1	ENST00000320574	
RAD50	Promoter	c.-159C>T	11.5	ENST00000378823	
ROS1	p.Glu268Lys	c.802G>A	10.6	ENST00000368508	
TCF3	p.Pro539Ser	c.1615C>T	37.4	ENST00000262965	
TERT	Promoter	c.-146C>T	33.3	ENST00000310581.5	II
TSC1	p.Glu446Gly	c.1337A>G	22.8	ENST00000298552	

Mutations discussed in the report are highlighted in bold; * nonsense mutations.

One month following the initial diagnosis, the patient presented to our institution and underwent a sentinel lymph node biopsy (SLNB); both isolated lymph nodes were negative for metastatic disease. Per patient preference, Mohs micrographic surgery was performed. Pathologic evaluation of the ear lesion confirmed negative surgical margins, supported by MART-1 immunostaining for melanocytes. He continued routine surveillance with clinical visits and physical exams every 3 months per NCCN guidelines. No molecular testing was performed at that time.

One year later, the patient developed a mildly uncomfortable lump on the left side of his face and neck. A contrast-enhanced neck computed tomography (CT) scan revealed a 19 mm × 16 mm hypodense structure at left level 2A, consistent with an abnormal lymph node (Figure 2C). Subsequent left neck ultrasound demonstrated a cystic, hypoechoic, and hypovascular lymph node measuring 14 × 10 × 14 mm^3^. A fine-needle biopsy of this lymph node demonstrated necrotic tissue with melanophages and melanin pigment deposition. Immunohistochemistry was positive for MART-1 and HMB-45, suggesting metastatic malignant melanoma. After the lymph node biopsy was performed, a staging PET CT was completed, confirming an FDG-avid left lower face soft tissue abnormality (SUV 6.5 with mediastinal blood pool SUV 2.3) without other metastatic sites. Brain MRI with contrast was also negative for metastatic disease. He was clinically staged as IIIB (rT3a N1 M0), and next-generation sequencing (NGS) was ordered on the lymph node biopsy.

**Figure 3:**
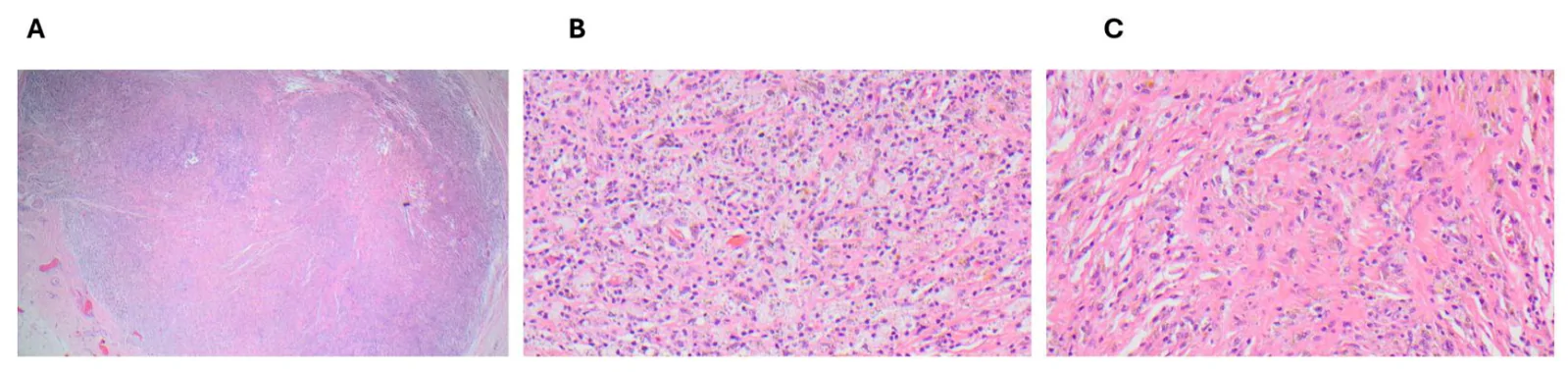
Histology of post-treatment resection at Month 16. (**A**) Lymph node with histiocytic inflammation, mild fibrosis, no viable tumor cells (20× magnification); (**B**) histiocytes at 200×, some with melanin pigment. (**C**) An area with fibrosis (200×).

Neoadjuvant immunotherapy was recommended per standard-of-care guidelines for stage III melanoma. The patient elected to receive two doses (administered 4 weeks apart) of a fixed-dose combination of nivolumab (anti-PD-1) and relatlimab (anti-LAG-3), followed by surgical resection, adhering to the protocol evaluated in the Phase II clinical trial NCT02519322 [26] (Figure 2A). This regimen was selected over ipilimumab plus nivolumab [27] to minimize treatment-related toxicity and optimize overall tolerability. The patient completed neoadjuvant therapy without experiencing any significant toxicities. Post-treatment surgical resection of the left parotid tail was performed, including seven lymph nodes and surrounding salivary gland tissue. Histopathologic evaluation demonstrated a pathologic complete response. Specifically, two lymph nodes showed treatment-related histiocytic inflammation and mild fibrosis, five additional lymph nodes were negative for malignant cells, one periparotid soft tissue nodule exhibited histiocytic inflammation, and the salivary gland parenchyma was free of tumor (Figure 3). Consequently, adjuvant immunotherapy was omitted, and the patient remains disease-free under active clinical surveillance.

NGS testing was also performed on this surgical specimen to further delineate the effectiveness of neoadjuvant immunotherapy. All molecular mutations previously identified in the Month 13 lymph node biopsy were undetectable at a 2% sensitivity threshold, confirming both a complete pathologic and molecular response.

## 3. Discussion

This case highlights the biological complexity of melanoma and the value of comprehensive genomic profiling. The patient achieved complete pathologic and molecular remission following neoadjuvant immune checkpoint blockade. The two *NF1* loss-of-function mutations and high tumor mutational burden likely contributed to his excellent response to immunotherapy. The outcome is in line with what was reported by Thielmann et al. [16]. The multicenter retrospective cohort study evaluated therapeutic response to first-line treatment with immune checkpoint inhibitors. Patients with NF1-mutated tumors (N = 266) achieved significantly improved median overall survival compared to those with wild-type tumors (N = 432); The median overall survival for the NF1-mutated cohort was not yet reached, compared to 25.82 months in the wild-type cohort. This exceptional sensitivity is attributed to the high TMB frequently found in NF1-altered malignancies, as in our patient.

Several genomic findings in this tumor are noteworthy. First, the lesion harbored a Class 3 *BRAF* Y472V mutation. Unlike classic V600 alterations, Class 3 *BRAF* variants are kinase-impaired and signal through an enhanced RAS-dependent mechanism to drive MAPK hyperactivation. Concurrent *NF1* loss may further amplify signaling through this pathway by prolonging RAS activation. Furthermore, the identification of two distinct *NF1* nonsense mutations suggests possible biallelic inactivation and supports *NF1* loss as a major cooperating driver. Notably, the co-occurrence of *NF1* loss with Class 3 *BRAF* mutations is well-recognized in cutaneous melanoma (Figure 1) [17].

However, the presence of two concurrent *KIT* mutations in this patient is highly unusual. The *KIT* K642E in exon 13 is located within kinase domain I, which is sensitive to TKI. With a variant allele frequency (VAF) of 44.8%, it represents the most dominant clone in this tumor (Figure 2D). Conversely, the *KIT* D816V variant in exon 17 represents a minor subclonal mutation detected at a low VAF of 5.2%. This activation-loop alteration stabilizes KIT in an active conformation to drive constitutive kinase signaling. While *KIT* D816V is a well-established primary resistance mechanism to first-generation TKIs (e.g., imatinib) across various malignancies, it may retain sensitivity to modern selective inhibitors such as avapritinib [28].

The identification of a resistance-associated *KIT* D816V mutation in a treatment-naïve tumor is particularly notable. This finding suggests that therapeutic resistance can stem from preexisting subclonal diversity rather than arising exclusively through treatment-induced selection. Should KIT-targeted therapy be considered in the future, pharmacological suppression of the dominant *KIT* K642E clone could promote the selective expansion of the preexisting D816V subclone, resulting in therapeutic escape and disease relapse.

## 4. Experimental Method

### 4.1. Next-Generation Sequencing

Targeted next-generation sequencing was performed using the QIAseq Targeted DNA Human Comprehensive Cancer Panel (QIAGEN, Germantown, MD, USA), starting with 60 ng of genomic DNA input from tumor or matched normal peripheral blood. DNA underwent enzymatic fragmentation, end-repair, and A-tailing, followed by adapter ligation to incorporate unique molecular identifiers (UMIs) to the 5′ ends. Target regions were then enriched via gene-specific primer extension, and final libraries were amplified using indexing primers alongside a universal primer. Amplified libraries were then purified, quantified, and pooled prior to sequencing on the Illumina NovaSeq X platform using a 1.5B Reagent Kit (150 bp paired-end reads; minimum depth of 20 million reads per sample). Raw FASTQ files were imported into QIAGEN CLC Genomics Workbench (version 21.0.4) for bioinformatic processing. Variant curation, annotation, and clinical interpretation were conducted using QIAGEN Clinical Insight (QCI) software (https://digitalinsights.qiagen.com/products-overview/clinical-insights-portfolio/qiagen-clinical-insight/na/qci-precision-insights/ accessed on 26 October 2026). Sequencing was performed on both the tumor specimen and matched normal tissue. Variants of somatic origin were presented in Table 1.

### 4.2. MSI Assay and TMB Calculation

MSI status was evaluated using the Promega MSI Analysis System (Version 1.2), a multiplex PCR-based fragment analysis. Genomic DNA was extracted from FFPE tumor tissue and matched normal tissue and was selectively amplified with fluorescein-labeled primers for eight microsatellite markers (NR-21, BAT-60, BAT-25, BAT-59, BAT-26, BAT-56, MONO-27, and BAT-52). Amplified DNA products were then detected by capillary electrophoresis fragment analysis. In tumor samples, the presence of new or shifted alleles relative to the germline allele sizes indicates microsatellite instability. Each of the eight microsatellites is scored for the presence or absence of MSI. Tumor samples were classified as MSI-High if instability was scored in two or more of the 8 markers. TMB calculation follows the formula: Number of nonsynonymous mutations/megabase sequenced. TMB ≥ 20 is scored as high with our NGS panel.

## 5. Conclusions

The co-occurrence of a primary *KIT* K642E driver mutation and a subclonal *KIT* D816V resistance variant in treatment-naïve melanoma has not been previously reported. This case underscores the importance of comprehensive molecular profiling, not only to identify actionable drivers but also to uncover low-frequency resistant subclones that may dictate treatment durability and clinical outcomes of future treatment.

## Data Availability

The original contributions presented in this study are included in the article. Further inquiries can be directed to the corresponding author.
